# Supplementary material for: Beyond Binary: A Machine Learning Framework for Interpreting Organismal Behavior in Cancer Diagnostics
Source: Biomedicines. 2025 Sep 30;13(10):2409. doi: 10.3390/biomedicines13102409 (PMC12561682; doi:10.3390/biomedicines13102409)
Supplement: Supplementary file 1 [file biomedicines-13-02409-s001.zip › biomedicines-3896366-supplementary.pdf]

Table S1. Minimal specifications and standardization guidelines for organismal biosensing assays.

Category	Parameter	Recommended Specification	Notes	References
Sample handling	Urine storage	4 °C ≤ 24 h or immediate freezing at −80 °C	Commonly reported practice in *C. elegans* and canine VOC assays	[32,61,74]
	Breath collection	Use inert bags (e.g., Tedlar®) or sorbent tubes; analyze within 6–12 h	Stability critical for VOC reproducibility	[83,90]
	Sweat/saliva	Immediate cooling (4 °C) and analysis within 12 h or freezing	Mostly pilot-level studies, not yet standardized	[81,82]
Environmental controls	Temperature	Maintain 20 ± 1 °C (*C. elegans*); 22–24 °C (canines/insects)	Temperature shifts alter behavioral baselines	[19,24,61]
	Humidity	40–60% relative humidity; avoid extremes	Impacts olfactory acuity and odor plume stability	[83,85]
Organism handling / training	*C. elegans* culture	NGM agar at 2–2.5% concentration; synchronized adults, 4 d at 20 °C	Plate preparation affects chemotaxis gradient stability	[32,74,79]
	Canine training	Standardized protocols with ≥ double-blind validation; monitor training drift (10–15% variability)	Training rigor directly linked to reproducibility	[61,80]
	Insect assays	Controlled antennal lobe exposure; standardized odor delivery	Neural readouts highly sensitive to odor plume dynamics	[24,84,85]
Assay-specific parameters	Chemotaxis assay (*C. elegans*)	Plates equilibrated ≥1 h; odorant spots with 1–2 µL VOC extract; CI formula standardized	CI = (A − B)/(A + B), where A = worms at attractant, B = worms at control	[32,74]
	Microfluidic arenas (*C. elegans*)	Use channels ≤100 µm; laminar flow verified before assays	Enhances gradient stability and reduces variability	[13,79]
	Scent presentation (canines)	Automated or blinded carousel systems; avoid handler cues	Mitigates expectancy bias	[61,80]
	Neural recordings (insects)	Use ≥16-channel electrode arrays; stimulus onset <200 ms	Enables high-resolution decoding of VOC signatures	[24,84]

Notes: This checklist summarizes minimal specifications for reproducible biosensing studies using *C. elegans*, canines, and insects. Recommendations are drawn from peer-reviewed methods papers where available, and from reported practices where formal standards are lacking. Abbreviations: VOC, volatile organic compound; CI, Chemotaxis Index.

Supplementary Note S2. Industry Update Source Materials

An Innovation Update was hosted by Hirotsu Bio Science in September 2024 and reported via the PR TIMES press release service. These materials are non–peer-reviewed and are cited here solely to provide transparency regarding industry-reported claims.

The press event included discussion of:

- Real-world N-NOSE data presented by the Japan Society of Nuclear Medicine PET Subcommittee.
- Ongoing large-scale clinical trials with the National Cancer Center of Japan.
- Expansion of detectable cancer types from 15 to 24.
- Integration of AI-based nematode behavioral image analysis, with a reported improvement in diagnostic AUC from 0.845 to 0.91.

Full details are available online at:

- Hirotsu Bio Science. “N-NOSE Innovation Update: Latest Developments in Nematode Cancer Screening.” PR TIMES. 20 September 2024. Available online: [https://prtimes.jp/main/html/rd/p/000000001.000000000.html] (accessed on 3 September 2025).

Readers should note that this press release is a company communication and has not undergone peer review; reported performance metrics should therefore be interpreted with caution. In line with best practice for transparency, these materials are included in the Supplement but not treated as peer-reviewed evidence
